# FTUC: A Flooding Tree Uneven Clustering Protocol for a Wireless Sensor Network

**DOI:** 10.3390/s17122706

**Published:** 2017-11-23

**Authors:** Wei He, Sebastien Pillement, Du Xu

**Affiliations:** 1 School of Information Engineering, Guang Dong University of Technology, Guangzhou 510000, China; du.xu@163.com; 2 Polytech-IETR, University of Nantes, rue Christian Pauc, 44300 Nantes, France; sebastien.pillement@univ-nantes.fr

**Keywords:** wireless sensor network (WSN), flooding tree, uneven clustering, unequal cluster, network

## Abstract

Clustering is an efficient approach in a wireless sensor network (WSN) to reduce the energy consumption of nodes and to extend the lifetime of the network. Unfortunately, this approach requires that all cluster heads (CHs) transmit their data to the base station (BS), which gives rise to the long distance communications problem, and in multi-hop routing, the CHs near the BS have to forward data from other nodes that lead those CHs to die prematurely, creating the hot zones problem. Unequal clustering has been proposed to solve these problems. Most of the current algorithms elect CH only by considering their competition radius, leading to unevenly distributed cluster heads. Furthermore, global distances values are needed when calculating the competition radius, which is a tedious task in large networks. To face these problems, we propose a flooding tree uneven clustering protocol (FTUC) suited for large networks. Based on the construction of a tree type sub-network to calculate the minimum and maximum distances values of the network, we then apply the unequal cluster theory. We also introduce referenced position circles to evenly elect cluster heads. Therefore, cluster heads are elected depending on the node’s residual energy and their distance to a referenced circle. FTUC builds the best inter-cluster communications route by evaluating a cluster head cost function to find the best next hop to the BS. The simulation results show that the FTUC algorithm decreases the energy consumption of the nodes and balances the global energy consumption effectively, thus extending the lifetime of the network.

## 1. Introduction

With the rapid development of wireless communications, integration of sensors, and embedded technology, the development of wireless sensor networks (WSNs) has become more and more attractive. A WSN [1,2,3] consists of a large number of sensor nodes (SNs) with wireless transmitter and data processing elements to perform monitoring of a, potentially large, sensed area. The increasing number of application domains for WSNs [4,5,6] has aroused great attention in academia and industry. As the network nodes are spread across a large area, they are mainly battery operated, leading to energy, computation, and resource limitations. It is then important to build energy efficient WSNs.

Topology control provides an effective way to cope with this challenge. In general, topology control refers to a set of technologies that can transform the underlying network topology to improve the system performances or reduce its costs [7]. Among all these methods, clustering [8] is an efficient and extensively used scheme to manage WSNs. In these networks, SNs are divided into several clusters, including a leader node named cluster head (CH) [9]. The CH is a bridge and creates the link between the nodes in its cluster and the rest of the network. Clustering has various significant superiorities over the classical scheme. Firstly, the SNs monitoring the environment send their data to their corresponding CH, which avoids the need for nodes to transmit large sets of data directly to the base station (BS) [3]. The CHs consume less energy for gathering data (referenced as intra-cluster communications) since they are close to the SNs [10]. Secondly, the CHs can be arranged in a multi-hop sub-network to forward data and save energy by avoiding long distance communications, named inter-cluster communications. However, due to the large forwarding data task, the CHs near the BS will see their battery depleted quicker. The zones including such CHs are called hot zones [11].

Unequal cluster-based routing (UCR) [12] introduces a competition radius to reduce the cluster sizes near the BS. Smaller clusters, with less SN members, decrease the CH energy consumption by lowering intra-cluster communications. Therefore, UCR reduces the hot zone problem to a certain extent. The UCR algorithm elects cluster heads depending on the competition radius, which depends on the relative node position in the network, leading to a possibly large number of CHs elected in the same area with similar position values. This is the unevenly distributed CH phenomenon. Due to this phenomenon, a new hot zones problem can be created in the network, and can also lead to uncovered nodes if they are too far from the BS. Also, UCR needs to calculate the competition radius; therefore, the global minimum and maximum distances between each node and the BS are needed. This is not realistic for large networks due to the amount of data required, and also for long distance transmissions that might be impossible for all nodes.

To solve these problems, we propose the flooding tree uneven clustering algorithm (FTUC). The main contributions presented in this paper are:

- A clustering algorithm enabling the CH election in large WSNs by building a tree network to convey the global minimum and maximum distance data to the BS.
- A way to balance the uneven distribution of CH by introducing referenced positions to elect CH, which balance the network load and prolong the network lifetime.
- The construction of unequal clusters to optimize the energy consumption of the overall network.
- A set of simulations to show the effectiveness of our approach, and to compare to state-of-art algorithms.

The remainder of this paper is organized as follows. We give a brief discussion on the related literature in Section 2. In Section 3, definitions, and models used in this work are introduced. In Section 4, the problems of current statements are introduced. The FTUC algorithm is presented in detail in Section 5. Comparisons against LEACH and UCR, two well-known routing algorithms, are presented in Section 6, to show the effectiveness of FTUC, while Section 7 concludes the paper.

## 2. Related Work

During the last decades, numerous protocols for wireless sensor networks have been proposed. These protocols can be classified into hierarchical, location-based, data-centric, network flow, and Quality of Service-aware routing [13]. Many energy efficient clustering and routing topologies have been proposed to prolong the network lifetime [8]. Low-Energy Adaptive Clustering Hierarchy (LEACH) [14] is one of the most popular hierarchical routing approaches for WSNs. LEACH uses a probability equation to select the CH, and the nodes join a specific cluster according to the Received Signal Strength Indication (RSSI) of CH neighbors. In the stable phase, i.e., when the clustering has been done, each CH forwards an aggregated data packet from all member nodes to the BS. At the periodical interval, the network rotates CHs to balance the network load and to preserve the energy of nodes. If LEACH is well deployed due to its effectiveness and simplicity, it suffers from the hot zone problem, and needs to support long-range communications for all nodes.

Threshold sensitive Energy Efficient sensor Network protocol (TEEN) [15] is a hierarchical protocol whose main goal is to cope with sudden changes in sensed values. In this protocol, sensed attributes hard thresholds (HT) and soft thresholds (ST) are defined. Then, TEEN constructs two-level CHs, which use HT and ST values, to determine whether to transmit data to CH and BS. When a node senses a value bigger than HT, it switches on its transmitter and send the value to its CH. When the CH receives data bigger than ST, it switches on its transmitter and transmits the value to the BS. By construction, TEEN reduces the CH load, but this approach is only suitable for sudden changes of measurements and is not suitable for periodical monitoring.

Hybrid Energy-Efficient Distributed clustering (HEED) [16] is a clustering algorithm with an explicit consideration of energy-efficiency. In HEED, cluster heads are elected depending on the node’s residual energy and intra-cluster communications cost. After a certain operational time, new CHs are uniformly elected for the network. Distributed Energy-Efficient Clustering Protocol (DCE) [17] elects cluster heads depending on a double-phase scheme. In the first phase, tentative CHs are elected depending on the node’s residual energy. In the second phase, the final cluster heads are chosen as nodes with the highest residual energy. HEED and DCE are both efficient algorithms to solve the problem of CH energy balancing. However, these protocols rely on the existence of nodes with bigger batteries, defining heterogeneous networks.

The Energy-Efficient Multi-level Clustering algorithm (EEMC) [18] is a hierarchical routing algorithm inspired by LEACH. EEMC is based on a multi-level clusters scheme. The SNs send their data to the lowest layer (level-n) CH, and afterwards, all the level-n cluster heads send data to level-(n–1) cluster heads. The level-1 CHs gather all the data and send them to the base station. It is an effective way to balance the cluster head node energy in a huge WSN structure, but the size of clusters is limited to reduce the intra-cluster communications. The level-1 CHs are also weaknesses for the network lifetime.

In order to solve the hot zone problem, Soro and Heinzelman [19] investigated an unequal clustering model for balancing the energy consumption of cluster heads in multi-hop sensor networks. The idea is to make the smallest clusters possible, thus reducing intra-communications costs, but this leads to a growing number of CHs. Then, clusters with a smaller size are built near the BS and bigger ones are situated further away from the BS. If the approach demonstrated is to be useful for managing the hot zone, this algorithm is based on static CHs with a large battery deployed in each cluster. The Unequal Cluster-based Routing (UCR) protocol [12] addresses this problem and proposes to compute the competition radius of nodes and builds clusters of different sizes depending on the node distance to the BS. This approach effectively solves the problem, and enables the creation of unequal clusters, while balancing the energy over time on different CHs.

However, in UCR, the CH election relies on the competition radius of the node, which depends on its relative global position. This can lead to electing several CHs in the same area due to the limited number of cluster heads. If some areas have a lot of CHs, the rest of the zone will build very large sized clusters with less CHs and even eventually without any. This is the unevenly distributed CHs phenomenon. This problem creates (i) isolated nodes that will waste a large amount of energy to send data directly to the BS; and (ii) some very large clusters, which will generate huge intra-cluster communications. In this situation, some nodes and CHs will die early due to their battery depletion and the whole network lifetime will decrease. Furthermore, the competition radius calculation requires knowing the global minimum and maximum distances from the nodes to the BS. This information is typically obtained through direct communications between nodes and the BS. Unfortunately, when dealing with large areas, some nodes are located too far away to communicate directly with the base station, and it is impossible to obtain the global minimum and maximum distance. In this case, most algorithms only consider the nodes that can directly communicate with the BS, reducing the area of the effective WSN. Moreover, it also wastes large amounts of energy on all nodes to send long distance information to the BS.

In this paper, we inherit the conception of the unequal cluster approach, but we further improve the energy-efficiency consumption of CHs, propose an approach suitable for large homogeneous networks, and offer a new solution to prolong the network lifetime by electing evenly distributed CHs throughout the network.

## 3. Definitions and Models

### 3.1. Network Model

In this work, without the loss of generality, we assume that a sensor network consists of N sensor nodes uniformly distributed in a circle area of an M meter radius. The BS is the center of the circle. S_i_ denotes the i-th sensor, and the corresponding sensor node set is S = {S_1_, S_2_, …, S_N_}, where |S| = N. We also assume that the sensors are homogeneous, i.e., they have the same capabilities, and each has a unique identifier i*d*. A node is assumed to communicate with at least one other sensor within its communication range, and can compute its approximate distance with another node based on the received signal strength (RSSI) measurement. Also, all the sensor batteries cannot be recharged and each sensor has power control capabilities to vary their transmitting/receiving power. This property is used to save energy locally once the node joins a cluster.

### 3.2. Node Energy Model

We employ the classical simplified node energy model [14,15,16]. In this model, a free space and a multi-path fades channels that are defined depending on the distance between the source and the destination nodes. The required energy for transmission depends on a threshold distance d_0_. If the distance from the source to the destination is less than d_0_, the free space model is used and the energy required by the amplifier to send data directly to the recipient is defined as $\varepsilon_{\mathrm{fs}}\left(\mathrm{pJ}/\left(\mathrm{bit}/m^{2}\right)\right)$. Otherwise, the multi-path $\varepsilon_{\mathrm{mp}}\left(\mathrm{pJ}/\left(\mathrm{bit}/m^{2}\right)\right)$ parameter is employed and represents the energy required to send data by using other sensors in a multi-hop way.

To transmit b-bits of data over a distance d_0_, the energy of transmission E_T_ is given by Equation (1).

(1) $$E_{T}=\{\begin{array}{cc} b\times E_{\mathrm{elec}}+\;b\times \varepsilon_{\mathrm{fs}}\times d^{2} & \mathrm{if}\;d<d_{0}\\ b\times E_{\mathrm{elec}}+b\;\times {\;\varepsilon }_{\mathrm{mp}}\times d^{4} & \mathrm{if}\;d>d_{0} \end{array}$$

In which E_elec_ (nJ/bit) is the sum of electrical energy required by the transmitter front-end to transmit one bit of data. The amount of energy expended by the front-end radio to receive b-bits of data is defined as:

(2) $$E_{R}=b\times {\;E}_{\mathrm{elec}}$$

In addition, we also assume E_sen_ to be the energy required to sense the environment, and E_com_ to be the energy needed for data aggregation.

### 3.3. Network Life Cycle

For any node i, we consider that each node has an initial energy E_ini_ that fulfills the requirements of the node for a life cycle. We define the expected node lifetime as LT_i_. Equation (3) then represents the relationship between E_ini_ and LT_i_.

(3) $$E_{\mathrm{ini}}=\overset{{\mathrm{LT}}_{i}}{\underset{t=0}{\sum }}\left\{\underset{j\epsilon N_{i}^{t}}{\sum }E_{R}^{t}\left(k_{\mathrm{ji}}\right)+{\;k}_{t}E_{\mathrm{com}}p_{t}E_{\mathrm{sen}}+\;\underset{l\in {\;Z}_{i}^{t}}{\sum }E_{T}^{t}\left(k_{\mathrm{il}},\mathrm{Dist}\left(s_{i},s_{l}\right)\right)\right\}$$

where N is the set of nodes that sends k_ji_-bit of data to node i at time t. Z is the set of nodes that receives k_il_-bit data from node i at time t. P_t_ is the number of measures performed by node i at time t. Dist(S_i_,S_l_) is the distance between the node i and the node l. k_t_ represents the data bit width to be processed by the node at time t.

We define the number of network surviving nodes at time t as LiveN(t), and we can then define the network lifetime, as in [20], as LT−1 as the time of first node death:

(4) $$\mathrm{LT}-1=\min\left\{{\mathrm{LT}}_{i}:i\in N\right\}$$

and LT−2 as the time where 70% of the nodes are dead:

(5) LT−2=max{t :liveN(t)≤0.7N}

### 3.4. Energy Consumption Balance

To evaluate the energy consumption balance across the network, we propose to use the energy means M_E_(t) and the energy variance $\sigma_{E}\left(t\right)$.

The mean residual energy is defined by Equation (6):

(6) $$M_{E}\left(t\right)=\frac{\sum_{i=1}^{N}E_{c_{i}}\left(t\right)}{N}$$

where $E_{C_{i}}\left(t\right)$ is the remaining energy of node i at time t. The variance of residual energy is given by:

(7) $$\sigma_{E}\left(t\right)=\frac{\sum_{i=1}^{N}{\left\{E_{c_{i}}\left(t\right)-M_{E}\left(t\right)\right\}}^{2}}{N}$$

From these definitions, it is obvious that a bigger M_E_(t) value represents more residual energy in the network, while smaller $\sigma_{E}\left(t\right)$ represents a more balanced node energy consumption.

## 4. Problem Statements

### 4.1. Unevenly Distributed Cluster Problem

**Figure 1:**
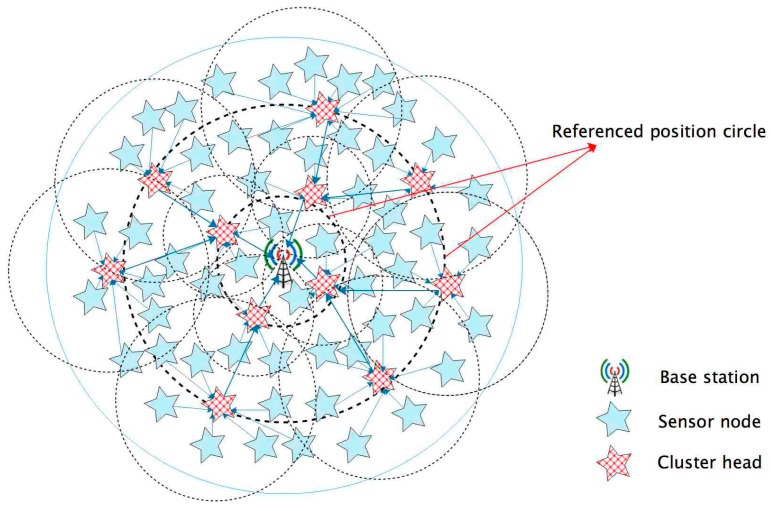
An overview of the FTUC protocol including the ’referenced position circles’ to elect evenly the CHs.

As mentioned above, the unequal clustering algorithms only elect CH depending on competition radius, leading to an uneven distribution of cluster heads. This problem then creates new hot zones in the network. In order to solve this issue, we introduce R_i_ as ‘referenced position circles’. As shown in Figure 1, the BS is the center of «all» circles, and the nodes that are close to a ‘referenced position circle’ have a bigger probability to be chosen as CHs. As soon as a referenced position circle has been set, the network elects cluster heads more evenly. In FTUC, we keep the competition radius concept, and choose the CHs with the most residual energy and the closest position to R_i_. The choice and the values for R_i_ are discussed in Section 4. After CH election, unequal size clusters are built depending on the competition radius.

### 4.2. Global Minimum and Maximum Distances Problem

For unequal clustering algorithms, the node competition radius evaluation is the most important parameter. In order to calculate this value, the nodes need three values: (i) Dist(S_i_,BS) as the distance between the node S_i_ and the BS; (ii) D_max_ as the longest path between a node and the BS; and (iii) D_min_ as the shortest distance between a node and the base station. D_max_ and D_min_ are global information calculated by the base station. If nodes exist that cannot communicate directly with the base station, it is therefore impossible to calculate the global minimum and maximum distances.

A way to solve this problem is by flooding the network [21] to transmit long-distance information to the base station using a multi-hop strategy. In this approach, each node broadcasts the messages until reaching the BS. In this situation, the broadcasting implies network congestion, consumes energy on each node, and loses lots of useful information, which can in turn make the communication channels unsafe.

To overcome this limitation, we propose a new approach called ‘flooding tree’. Above all, we define the base station as a unique root node at the center of the network. Then, the root node broadcasts information to its neighboring nodes. In an iterative process, when the node receives the information it acknowledges the reception and broadcasts the information to its neighborhood until the end of the network. Finally, a tree type sub-network is constructed taking into account the fastest path in the network.

In the built tree, the nodes between the end of the tree and the root node represent the route distance information. Then, the root node has to compare each depth of the tree branch to find the global minimum and maximum distances. If the network is static (there are no dynamic nodes), this phase has to be performed only one time at the first iteration of the algorithm.

## 5. FTUC Algorithm

**Figure 2:**
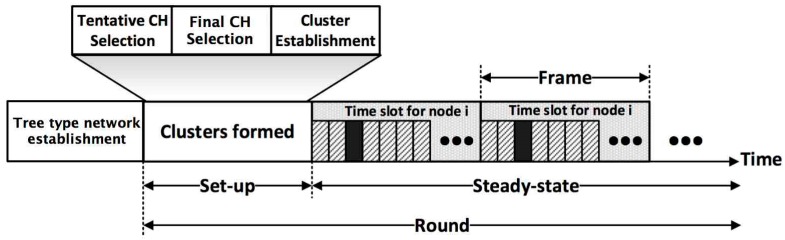
Timeline showing the operations of FTUC: at the first iteration, it builds a tree type sub-network. Then, periodically, it forms the clusters, and progresses to the steady state, where sensing and data transmissions are performed.

The FTUC algorithm is divided into three operational phases as depicted in Figure 2. In the flooding tree phase, the base station broadcasts the ‘build tree’ message to all nodes to set up a tree type sub-network. After the tree-type network construction, all distance messages are transmitted through this sub-network and the BS calculates the global distance values. In the clustering phase, the network elects the CHs and builds the unequal clusters. In the stable phase, the network is a classical time division multiple access (TDMA) WSN, in the sense that each node measures its environment and sends its data to the BS through the CH inter-cluster route. The last two steps (i.e., clustering and stable phases) are re-iterated periodically in order to elect new CHs and to balance the energy consumption over the nodes.

### 5.1. Flooding Tree Subnetwork

In this phase, the objective is to find the global distances in the network, without flooding all the WSN. We then rely on the creation of a tree along which information is propagated. This leads to energy savings, safe operations, and supports large area networks. Even if this approach is time-consuming, it has to be noticed that it is performed once for static networks. The details of the algorithm are shown in the Algorithm 1 pseudo-code. At each step of the algorithm, a time-out mechanism permits one to not stay blocked waiting for a message, not shown for the sake of clarity.

In the network, the base station is the root node. The nodes at the end of the network are called leaf nodes. The other nodes are called branched nodes. At the beginning, all the nodes are initialized as leaf nodes and then set the variable ‘leaf = true’ (line 8), except the base station, which is identified as the root note (i.e., id = id root), Algorithm 1.

**Algorithm 1** Building the tree sub-network**Input: id root** **Output: leaf**1: id = getId()13: if (type == T1) and (once == false) then2: if (id == id root) then14:  once = true3:  leaf = false15:  message = (T2, id)4:  once = true16:  send(message, rid)5:  message = (T1, id)17:  message = (T1, id)6:  send(message, *)18:  send(message, *)7: else19: end if8:  leaf = true20: if (type == T2) then9:  once = false21:  leaf = false10: end if22:  end if11: while (true) do23: end while12:  (type, rid) = read()

At the beginning of the process, the root node broadcasts ‘T1 information’ to all nodes within its radio range (line 5). When the neighboring node first receives the message, it is set as ‘once = true’ to indicate that it has received the message. This node then sends back ‘T2 information’ to the source of the received ‘T1 information’ to acknowledge the reception (line 13—18). This process enables one to create a path between the nodes throughout the network. If a node receives the ‘T2 message’, then it is not a leaf node, and is set as the variable ‘leaf = false’, indicating that it is a branched node. Parallel to this acknowledgment, the node broadcasts the ‘T1 information’ to its neighborhood until reaching the end of the network. If a node receives the ‘T1 message’ twice, it simply discards the message. As we only monitor the first received T1 message, the tree branches are created using the fastest paths. When all the nodes have received the ‘T1 information’, meaning that the tree network has been built, all the nodes that have not received the ‘T2 message’ are leaves. In this way, the tree network is created with a unique path from all nodes to the BS.

**Figure 3:**
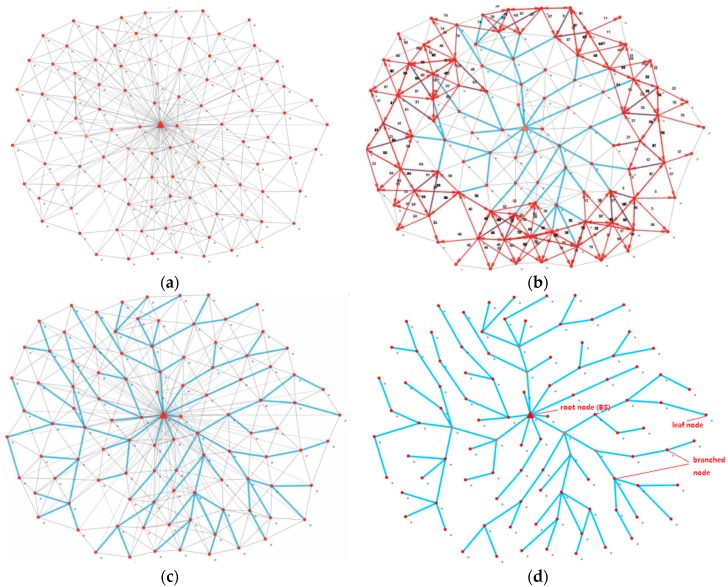
Flooding tree algorithm phases to build a tree type sub-network. The flooding phase enables one to create a unique and fast path between all nodes in the network. (**a**) Network initialization; (**b**) Running flooding tree algorithm; (**c**) Build tree type network; (**d**) Network finished.

The process of tree sub-network creation is illustrated in Figure 3. Figure 3a shows the network initialization process and the propagation of T1 messages is shown in Figure 3b. Figure 3c shows the network at the end of the broadcasting phase, while the finalized tree is shown in Figure 3d.

Finding the distances values of each branch of the tree sub-network is then obvious. The leaf nodes send the ‘distance information’ to their previous nearest branched node (the one from which they received the T1 message). This node compares all the distance values it receives and finds the smallest and the greatest ones. It then transmits these values to the next branched node until the root node. Finally, the root node receives the local minimum distance value of each branch of the tree network and can select the global minimum and maximum values. These values are then redistributed to all nodes through the tree, for the competition radius computation.

Our algorithm is more reliable than its counterparts to determine the maximum and minimum values while transmitting a smaller number of messages, as discussed in Section 5.

### 5.2. Building Cluster Phase

Once the minimum and maximum values are distributed in the network, the algorithm starts to create the unequal clusters. This process begins by randomly electing tentative cluster heads, and all nodes calculate their weight value and competition radius. Secondly, the tentative cluster heads choose the node with the biggest weight value as the final cluster head in its own competition radius range. Finally, the final CHs broadcast a ‘join cluster’ message to build their own clusters, and then send the ‘search cluster’ message in order to build the inter-cluster route. The clustering algorithm is executed as follows:

Step 1: calculate weight value and competition radius

After receiving D_max_ and D_min_ values from the BS, each node uses Equation (8) to calculate its competition radius R_competition_.

(8) $$R_{\mathrm{competition}}=\left(1-C\frac{D_{\max}-\mathrm{Dist}\left(S_{i},\mathrm{BS}\right)}{D_{\max}-D_{\min}}\right)R_{C}$$

where Dist(S_i_,BS) is the distance between the current node and the base station, R_c_ is the communication range of the radio front-end, and C is a control parameter belonging to [0, 1]. This parameter permits one to control the size of the created cluster depending on the distance to the BS. For example, if C is set to $\frac{1}{4}$, R_competition_ varies from $\frac{3}{4}$R_c_ to R_c_ according to the distance between S_i_ and the base station.

In order to evenly distribute the CHs, we propose the ‘reference position circle’ R_i_, evaluated as:

(9) $$R_{i}=\left|\sin\frac{\mathrm{Dist}\left(S_{i},\mathrm{BS}\right)\times 2\pi }{R_{0}}\right|$$

where R_0_ is the control parameter of the conferenced position circles. As R_i_ divides the total area of sensing into several zones, it should be chosen according to M and R_c_. The trade-off is made since all the nodes should be able to communicate with a CH near a position reference circle, and the number of circles should not be too high to evenly distribute the CHs in the area.

At this stage, each node calculates its weight value (W0):

(10) $$W0=C1\times R_{i}+C2\times E_{c}$$

where C1 and C2 are control parameters that enable one to make a trade-off between the node residual energy, E_c_, and its distance to the ‘reference position circle’. For example, if R_0_ in Equation (9) is set to 200 m, when a node is at Dist(S_i_,BS) = 50 m or Dist(S_i_,BS) = 150 m from the BS, R_i_ is at its maximum value of 1. Considering nodes with the same residual energy, the closest node to R_i_ will have a greater chance to be elected as a CH.

Step 2: Randomly Generate Tentative CH

At this step, each node randomly generates a number Q, and if Q < T, the node is set as a tentative CH. The threshold T enables one to control the number of tentative CHs in this phase. For example, if T = 0.08, then 100 nodes will elect eight tentative CHs.

Step 3: Final CHs choice

Each tentative CH then broadcasts an ‘id message’ in its competition radius R_competition_, and when regular nodes receive this message, they send back their own W0 value. Afterward, the tentative CH compares each received W0, including the one from itself, and chooses the node with the biggest value as the final CH, denoted as fCHi for cluster i. Finally, the elected fCHs broadcast the ‘join cluster message’ in their zone of influence (i.e., its competition radius).

Step 4: Clustering phase

When the nodes receive a ‘join cluster message’, they choose the nearest CH according to the RSSI received signal strength. The unequal sizes of clusters are determined at this step. Each node also adapts its transmitting power according to its distance to the fCH, in order to save energy.

Step 5: Isolated nodes

It is possible that a CH competition radius that does not cover some areas arises, and then, nodes cannot receive the ‘join cluster’ message. These nodes are then considered as isolated, and will send their data directly to the base station. It is obvious that this situation should be avoided if possible.

### 5.3. Inter-Cluster Communication

Once the final cluster heads are elected, they need to establish a multi-hop communications route to transmit the data to the base station. In order to do so, each final cluster head (fCHi) needs to find the best next hop (fCHj) until reaching the base station.

In this process, each fCH broadcasts a ‘search cluster message’ using its maximum radio communication range. Then, for all received messages j, fCHi calculates the cost function p(i,j) by using Equation (11).

(11) $$p\left(i,j\right)=\alpha \times \frac{1}{E_{c}}+\beta \times \mathrm{Dist}\left(\mathrm{fCHj},\;\mathrm{BS}\right)+\gamma \times \mathrm{Dist}\left(\mathrm{fCHi},\;\mathrm{fCHj}\right)$$

where α, β, γ are weighting factors that should respect the following condition: α+β+γ=1. Finally, each final cluster head uses a greedy algorithm to choose the minimum cost function value fCHj as the next hop.

For example, if fCHi needs to find its optimal next-hop, and there are three fCH (fCHa, fCHb, and fCHc) in its communication range, and Dist(fCHi,BS) = d1. Firstly, fCHi broadcasts a ‘search cluster message’ to fCHa, fCHb, and fCHc including the d1 value. When those fCHs receive this message they compare their own Dist(fCH,BS) to d1. If we consider that fCHa and fCHb are closer to the BS, then they start to compute Dist(fCHi,fCHa) and Dist(fCHi,fCHb), respectively. fCHc cannot be chosen as the next hop since it is farther from the BS. In order to choose between fCHa and fCHb, they use their residual energy and use the calculated distance to fCHi to compute the cost function p(i,j) (using Equation (11)) and send their results to fCHi. If we consider that the p(a,i) value is less than p(b,i), then fCHi will choose fCHa as the next-hop.

As can be seen from this process, there is a greater probability for a cluster head to be the next hop node when it has more residual energy, it is closer to the BS, and the distance between the two cluster heads is short.

Using simulations, we determined that the best compromise in a network lifetime is obtained with α=0.4, β=0.3, and γ=0.3. Giving a highest priority to the cluster head node residual energy enables one to have a better balanced communications load.

### 5.4. Stable Communication Phase

After the cluster and inter-cluster routes creations, the wireless sensor network goes into the stable communication phase. Utilizing a TDMA schedule, each SN monitors the environmental data and sends them to its cluster head. The fCH gathers the data from all its member nodes and forwards the data to the next-hop cluster head node until the base station receives all the data.

After an iteration time T_R_, the wireless sensor network releases all the cluster structures, and the cluster phase is re-executed.

## 6. Simulation and Analysis

In this section, we provide several simulation experiments to compare FTUC with the classical WSN LEACH [14] and UCR [12] routing algorithms. We first start by evaluating the parameters of our algorithm to find their optimal values, and we then make several comparisons with state-of-art algorithms.

In order to achieve a higher level of confidence, we conducted 20 experiments in each scenario. The network life times (LT-1 and LT-2) and percentage of alive sensor nodes were used as evaluation standards for WSN longevity.

We used the Cupcarbon tool [22] for our simulations. CupCarbon is an open-source simulator specifically designed for Smart City and Internet of Things Wireless Sensor Network (SCI-WSN) evaluations. It enables a visual analysis of WSN and permits a fast evaluation of new approaches. For the experiments, we assume a WSN with N = 100 sensor nodes, distributed uniformly in an M = 200 m, and the base station is the center of the sensing field (coordinate (0,0)). Each sensor monitors its environment once, sends 1-bit data every 10 s, and has E_init_ = 100 Joules of initial energy. The transmission range of each sensor is set to be R_c_ = M = 200 m. This value has been chosen to make fair comparisons with the LEACH and the UCR algorithms that require direct communications between each node and the BS.

The technological parameters used for all the WSN are resumed in Table 1; these values have been chosen as they are used in the literature [14].

**Figure 4:**
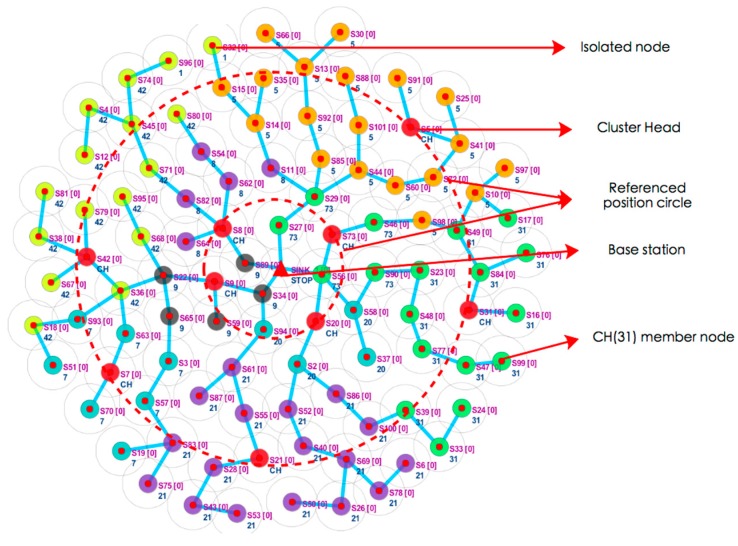
Cupcarbon simulation of FTUC. Cluster heads are evenly distributed in the network, and unequal clusters are created.

The Cupcarbon simulation result of FTUC is shown in Figure 4. As explained above, a tree network is first built, and CHs are elected among the nodes. As can be seen, the fCHs (in red on the figure) are uniformly distributed near the reference position circles. The remaining nodes then choose a cluster (represented by a same color in the figure). Smaller clusters are created near the BS and bigger ones are built further away from the BS.

### 6.1. FTUC Parameterization

In the first part of the experiments, we evaluate the impact of the FTUC parameters on the lifetime of the network. The initial values of the parameters are set as C = 0.8, C1 = 0.8, C2 = 0.2, T = 0.1, R_0_ = 100, α = 0.4, β = 0.3, and γ = 0.3, and the iteration time T_R_ = 300 s.

**Figure 5:**
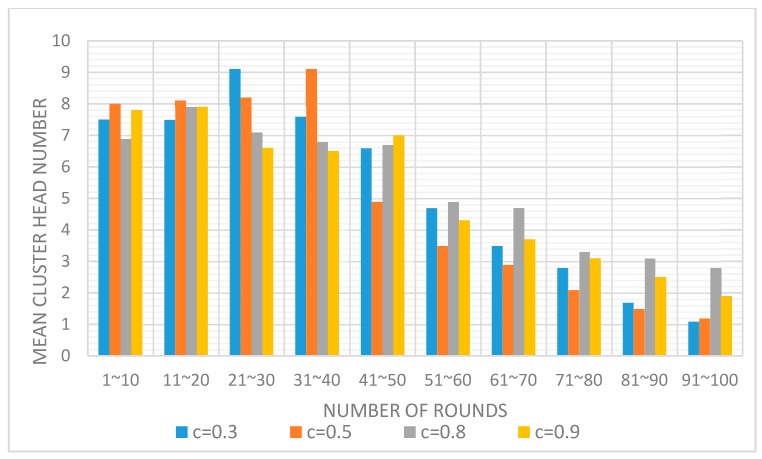
Number of elected CH per 10 rounds, depending on the competition radius calculation.

The C parameter controls the computation of the competition radius and determines the number of fCH in the network. We evaluated (Figure 5) the average number of cluster heads elected every 10 rounds of the network lifetime, depending on different values of C.

As can be seen, at the beginning of the network lifetime, each node has enough energy to elect a sufficient cluster heads number. However, after 50 rounds, the number of cluster heads decreases dramatically as the result of the apparition of dead nodes. Then, a lower number of nodes leads to the generation of less tentative CHs, and these tentative CHs have a higher probability of choosing the same node as the final cluster head in an adjacent area, creating bigger clusters. As can be seen, when C = 0.8, the number of CHs is more uniformly elected. This value is a good trade-off between the size of the competition radius of each node and the number of elected fCH. This number has a great impact on the overall distribution of possible CHs throughout the network.

**Figure 6:**
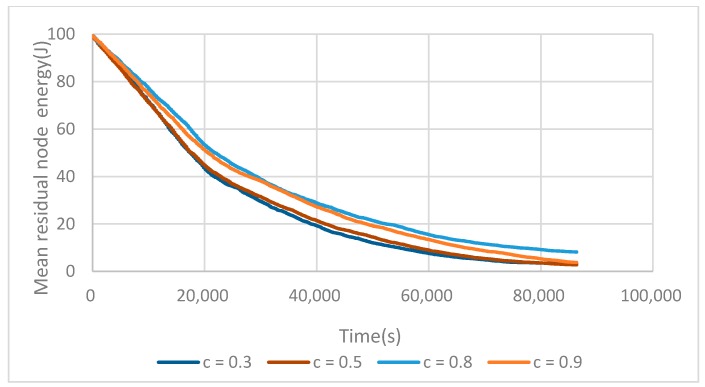
Evolution of M_E_(t) depending on the C value.

We evaluated the mean residual energy depending on C. In Figure 6, we can find that as the C value increases up to 0.8, M_E_(t) increases too. If C continues to grow, the competition radius near the BS becomes very small, resulting in very large clusters being located farther away. These large clusters in turn see their fCH dying prematurely.

Finally, it can be seen that C = 0.8 is the best compromise between the number of elected fCHs and the mean residual energy and has been chosen for the rest of the experiments.

**Figure 7:**
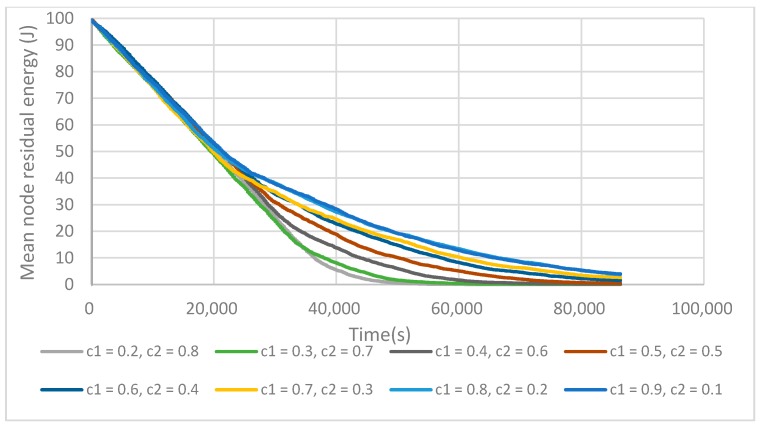
C1 and C2 value influences on the mean residual energy of the network. Well-positioned fCHs enables one to prolong the network lifetime.

The C1 and C2 parameters enable one to make a trade-off during the CH election between the relative position of the node to the reference circle and its residual energy (see Equation (6)). These parameters should respect the condition C1 + C2 = 1. Figure 7 shows the relation between the mean residual energy of the network and C1 and C2. As can be seen with the increase of C1, the network lifetime also increases. We can see that a bigger C1 values makes the position factor more important than the energy factor to prolong the network lifetime. The results in Figure 7 show that when a final cluster head is elected with a good position, it wastes less energy on inter-cluster communications as the CHs are evenly placed in the network. Therefore, it can prolong the network lifetime efficiently.

**Figure 8:**
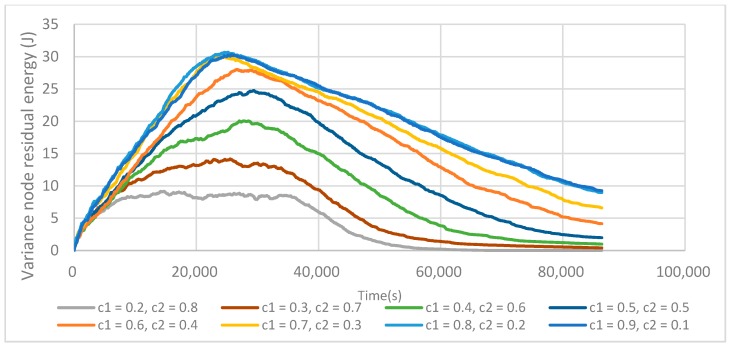
Variance of residual energy depending on C1 and C2. Lowering C2 increases σ_E_(t) as the residual energy of nodes has less impact on the fCH selection.

Figure 8 reports the influence of C1 and C2 values on the energy variance (Equation (7)), but it appears as a different trend when compared to what was previously thought.

From this point of view, increasing C1 also increases σ_E_(t). This comes from the fact that the residual energy of each fCH decreases faster, thus increasing the network energy variance. When C1 continues to increase from 0.8 to 0.9, M_E_(t) does not increase anymore but the variance does. So, it means that even if fCHs are well placed, it is not possible to prolong the network lifetime. C1 = 0.8 and C2 = 0.2 thus represent the best compromise between the fCH placement and its residual energy.

**Figure 9:**
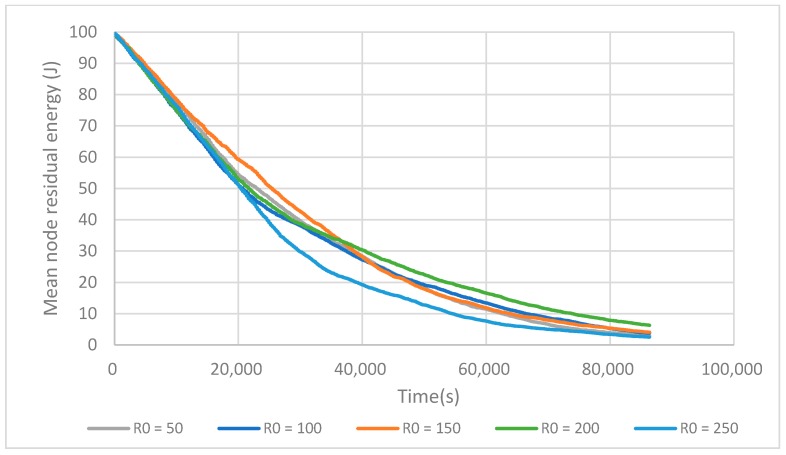
Evolution of the mean residual energy depending on the referenced position circles.

As seen above, selecting well-placed fCHs is a good approach to prolong the network lifetime. The positions of R_i_ are then crucial. We then investigate R_0_, which determines the position of the reference circles. From Figure 9 it can be seen that when R_0_ = 200 m, the network has its maximum lifetime. In the experimental setup, this value enables one to construct enough small size clusters near the BS to save energy in intra-cluster communication, and construct an optimal number of node clusters in the zone located further away.

**Figure 10:**
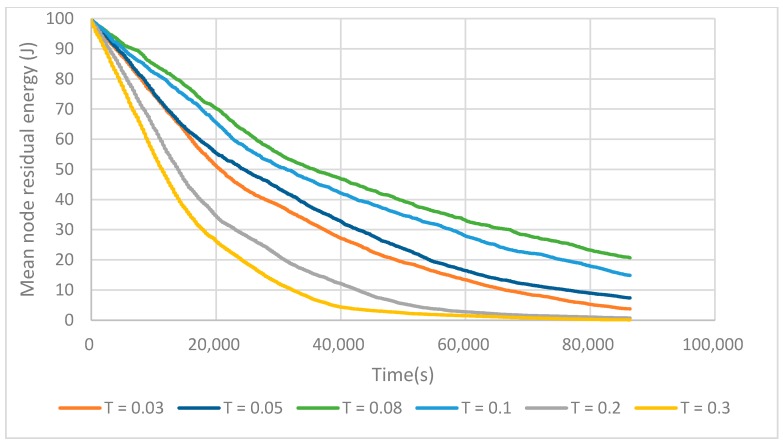
T value impact on network lifetime.

The T value determines the number of tentative cluster heads elected. As shown in Figure 10, as the value of T increases up to 0.08, the network lifetimes increase gradually. This is due to the fact that increasing the number of tentative cluster heads enables the election of a sufficient number of final cluster heads that can cover all the sensing area. Otherwise, there are a lot of isolated nodes, which wastes lots of energy when communicating directly with the BS. With the increase of T from 0.08 to 0.3, the network lifetime decreases dramatically. This is because electing too many tentative cluster heads means that the nodes waste too much energy on the building cluster phase.

**Figure 11:**
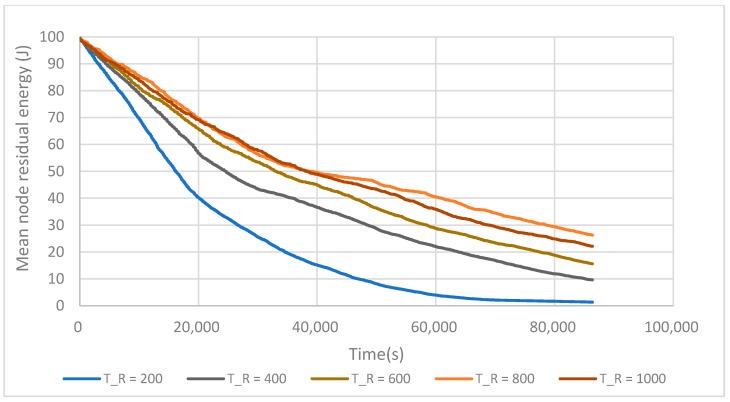
Impact of the iteration time T_R_ on the network LT-1 lifetime.

After the clustering phase, the network goes into the stable phase, in which CHs consume a large amount of energy. The iteration time T_R_ enables one to balance the network energy load by changing, if required, the CHs. Figure 11 shows the relation between the iteration time and LT-1. When the iteration time is short, for example, 200 s, the network lifetime is also short. This is due to the fact that the network wastes too much energy on building clusters. With the iteration time T_R_ = 800 s, the network lifetime reaches a maximum value. But when the iteration time continues to increase, the network lifetime decreases. The reason for this is that spending too much time between two iterations leads to fast CH battery depletion and the loss of these nodes. The iteration time is set to T_R_ = 800 s in the rest of this work to enable comparisons between all the algorithms.

The optimal values of parameters for FTUC are then resumed in Table 2.

### 6.2. Analysis of Cluster Creation

To improve network energy efficiency, UCR and FTUC use the creation of unequal clusters, making this phase more complex compared to LEACH. In FTUC, when the network is in the form of a tree structure, N nodes need to broadcast ‘T1 messages’, and then broadcast an N×T ‘ID message’ in the N×T temporary cluster head election stage. When K final cluster heads are elected, K ‘join cluster message’ are then broadcasted. Finally, the N–K cluster member nodes broadcast an N–K ‘Join cluster message’. Therefore, the message overhead for the whole process is: N + N×T + K + N–K = N(T + 2) messages, and the complexity for the cluster formation in FTUC is in O(N).

In UCR [12], the clustering phase adds up (2T + 1)N messages per round and the complexity is also in O(N). As can be seen, although we add the tree type network construction, the network complexity does not increase.

In FTUC, to determine the global minimum and maximum distance values, each node sends and receives one message, assumed to be of a k bit width. Under the presented conditions above, the free space model is leveraged for the communications, and the network energy consumption is as follows:

(12) $$\begin{array}{c} E_{1}=\sum_{i=1}^{N}\left\{E_{R}+E_{T}\left(d_{ij}\right)\right\}\\ \to =\sum_{i=1}^{N}\left\{k\times E_{elec}+\left(k\times E_{elec}+k\times \varepsilon_{fs}d_{ij}^{2}\right)\right\}\\ \to =\sum_{i=1}^{N}\left\{2k\times E_{elec}+k\times \varepsilon_{fs}d_{ij}^{2}\right\} \end{array}$$

In UCR, it is considered that each node can directly communicate with the base station to calculate the distance values. According to the experimental setup, nodes within a radius of d_0_ meters centered on the base station communicate using the free space mode. All the other nodes are considered as using the multi-hop way model. The network energy consumption is then:

(13) $$\begin{array}{l} E_{2}=\sum_{i=1}^{a}\left\{E_{T}\left(d_{iBS}\right)\right\}+\sum_{j=1}^{b}\left\{E_{T}\left(d_{jBS}\right)\right\}\\ \to =\sum_{i=1}^{a}\left\{k\times E_{elec}+k\times \varepsilon_{fs}d_{iBS}^{2}\right\}+\sum_{i=1}^{b}\left\{k\times E_{elec}+k\times \varepsilon_{mp}d_{jBS}^{4}\right\} \end{array}$$

where a is the number of nodes in the circular area near the base station, and b = N–a is the number of remaining nodes.

So, we can see (comparing E_2_ and E_1_) that the FTUC algorithm requires less energy consumption than UCR to find the maximum and minimum distance values.

### 6.3. Energy Efficiency

**Figure 12:**
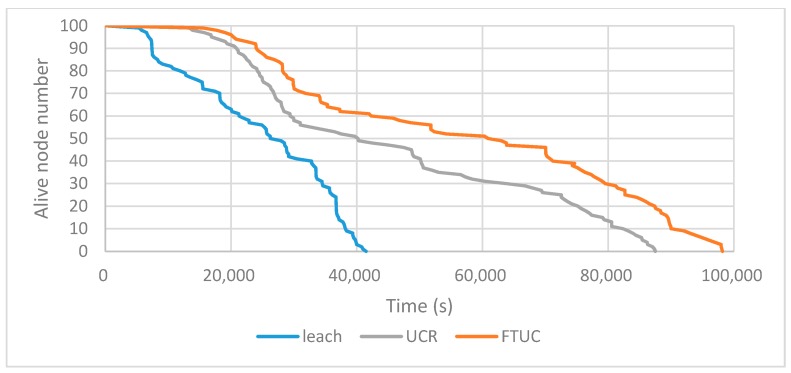
Network alive node numbers depending on time using LEACH, UCR, and FTUC.

Figure 12 shows the evolution of LiveN(t), the alive node numbers in FTUC, UCR, and LEACH during the network's lifetime. In the same operating conditions, FTUC shows its ability to increase the lifetime of the network by evenly distributing the CHs and by creating unequal clusters.

We extracted the LT-1 and LT-2 evaluation parameters from these experiments as resumed in Table 3. It shows that FTUC has an increased lifetime compared to LEACH and UCR (both according to LT-1 and LT-2 indicators). It shows up to 16% improvement over UCR in LT-1, because the tree-type network decreases the energy consumption required to obtain the global distance information, and no long-distance communications are needed. Furthermore, the network lifetime of FTUC is up to 24.7%, which is more than that of UCR in LT-2, due to a better distribution of CHs among the network, this in turn providing a better balance in the energy consumption of nodes. The improvements over LEACH confirm that creating unequal clustering is an efficient way to improve energy efficiency. Furthermore, LEACH cannot avoid long distance communications between all the nodes and the BS.

**Figure 13:**
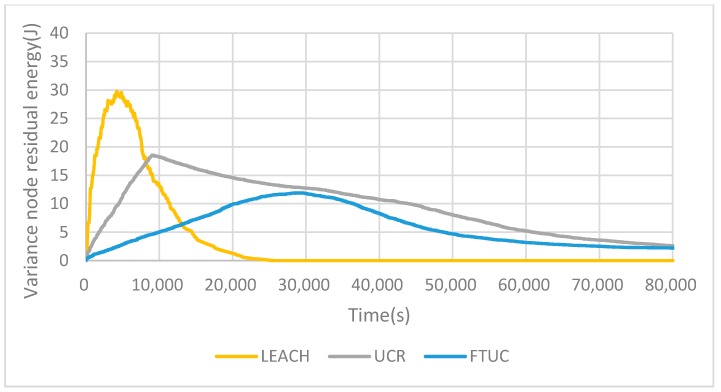
Variance of residual energy for each network. FTUC enables one to have a better repartition over time, increasing the network lifetime.

Figure 13 shows the energy variance of nodes depending on the routing algorithm. As can be seen, FTUC’s variance is lower than that of LEACH and UCR. This means that the FTUC algorithm decreases the energy consumption and balances the network energy consumption more efficiently.

## 7. Conclusions

Due to the uneven energy consumption in wireless sensor networks, a flooding tree uneven clustering protocol (FTUC) is proposed based on the conception of an unequal cluster protocol.

FTUC is designed to obtain the global minimum and maximum distance values of the nodes to the base station through a tree type sub-network, and introduces referenced circles to elect CHs more evenly throughout the network. Through the weight value and competition radius, the CHs are evenly distributed and unequal clusters are built. In order to avoid long-distance communications, an inter-cluster route is constructed according to the distance needed to reach the BS and the remaining energy of CHs. Therefore, FTUC has a lower energy consumption compared with LEACH and UCR in the cluster formation stage and during the inter-cluster communication stage. Furthermore, FTUC solves the energy consumption balance problem for each node. The simulation results show that the FTUC protocol reduces the energy consumption of the network effectively and prolongs the lifetime of the network.

As FTUC uses a very efficient strategy to compute the competition radius, we are working on its evolution to support dynamic networks. In this model, new nodes can appear (or disappear) during the network lifetime and should be managed efficiently. In such a dynamic network, security is now a really important property that must be taken into account. In that way, new strategies need to be integrated, even in FTUC. Our work can then benefit from using new approaches like the one presented in [23], and this requires further work.

## Figures and Tables

**Table 1:** Simulation parameters for the networks.

Parameter	Value
$E_{\mathrm{elec}}\left(\mathrm{nJ}/\mathrm{bit}\right)$	50
$\varepsilon_{\mathrm{fs}}\left(\mathrm{pJ}/\left(\mathrm{bit}/m^{2}\right)\right)$	6
$\varepsilon_{\mathrm{mp}}\left(\mathrm{pJ}/\left(\mathrm{bit}/m^{2}\right)\right)$	0.0011
$d_{0}\left(m\right)$	87
$E_{\mathrm{sen}}\left(\mathrm{nJ}/\mathrm{bit}\right)$	0.001
$E_{\mathrm{com}}\left(\mathrm{nJ}/\mathrm{bit}\right)$	0.001

**Table 2:** Optimal parameter values for FTUC.

Parameter	Value
C, C1, C2	0.8, 0.8, 0.2
T, R_0_	0.08, 200
T_R_ (s)	800
α, β,γ	0.4, 0.3, 0.3

**Table 3:** Evaluation of lifetime LT-1 and LT2 for all three algorithms.

	LT-1 (s)	LT-2 (s)	Improved of LT-1 (%)	Improved of LT-2(%)
LEACH	5420	34,510	186.3	130.6
UCR	13,380	63,810	16.0	24.7
FTUC	15,520	79,510		
